# Association of the low-density lipoprotein cholesterol/high-density lipoprotein cholesterol ratio and concentrations of plasma lipids with high-density lipoprotein subclass distribution in the Chinese population

**DOI:** 10.1186/1476-511X-9-69

**Published:** 2010-07-09

**Authors:** Li Tian, Yinghui Liu, Yang Qin, Shiyin Long, Yanhua Xu, Mingde Fu

**Affiliations:** 1 Department of Biochemistry and Molecular Biology, West China School of Preclinical and Forensic Medicine, Sichuan University, Chengdu 610041, Sichuan, China; 2 Department of Biochemistry and Molecular Biology, University of South China, Hengyang 421001, China; 3 Chengdu Hoist Biotechnology Co., LTD, Chengdu 610075, China; 4 Laboratory of Endocrinology and Metabolism, West China Hospital, Sichuan University, Chengdu 610041, Sichuan, China

## Abstract

**Background:**

To evaluate the relationship between the low-density lipoprotein cholesterol (LDL-C)/high-density lipoprotein cholesterol (HDL-C) ratio and HDL subclass distribution and to further examine and discuss the potential impact of LDL-C and HDL-C together with TG on HDL subclass metabolism.

**Results:**

Small-sized preβ_1_-HDL, HDL_3b _and HDL_3a _increased significantly while large-sized HDL_2a _and HDL_2b _decreased significantly as the LDL-C/HDL-C ratio increased. The subjects in low HDL-C level (< 1.03 mmol/L) who had an elevation of the LDL-C/HDL-C ratio and a reduction of HDL_2b_/preβ_1_-HDL regardless of an undesirable or high LDL-C level. At desirable LDL-C levels (< 3.34 mmol/L), the HDL_2b_/preβ_1_-HDL ratio was 5.4 for the subjects with a high HDL-C concentration (≥ 1.55 mmol/L); however, at high LDL-C levels (≥ 3.36 mmol/L), the ratio of LDL-C/HDL-C was 2.8 in subjects, and an extremely low HDL_2b_/preβ_1_-HDL value although with high HDL-C concentration.

**Conclusion:**

With increase of the LDL-C/HDL-C ratio, there was a general shift toward smaller-sized HDL particles, which implied that the maturation process of HDL was blocked. High HDL-C concentrations can regulate the HDL subclass distribution at desirable and borderline LDL-C levels but cannot counteract the influence of high LDL-C levels on HDL subclass distribution.

## Introduction

Lipid abnormalities have long been suspected to contribute to atherosclerosis (As); there is overwhelming evidence [1,2] that an elevated low-density lipoprotein cholesterol (LDL-C) concentration in plasma is atherogenic, whereas a higher high-density lipoprotein cholesterol (HDL-C) level is cardioprotective [2-4]. A series of studies suggested that the use of the ratio of LDL-C to HDL-C is superior to the use of HDL-C or LDL-C alone [5] and that the ratio of LDL-C/HDL-C may provide better risk assessment by concurrently accounting for both atherogenic and protective lipid fractions.

The cardioprotective effect of HDL has been largely attributed to its role in reverse cholesterol transport (RCT), wherein excessive cholesterol is conveyed from peripheral tissues to the liver and steroidogenic organs. HDL encompasses a heterogeneous class of lipoproteins. Differences in the quantitative and qualitative content of lipids, apolipoproteins (apos), and enzymes result in the presence of various HDL subclasses, which are characterized by differences in size, shape, charge and antigencity [6]. Using two-dimensional gel electrophoresis coupled with immunoblotting, HDL can be divided into large, cholesterol-rich (HDL_2a _and HDL_2b_), small, lipid-poor (HDL_3c_, HDL_3b_, HDL_3a_, and preβ_1_-HDL) and preβ_2_-HDL. It has been postulated that RCT indeed is the smallest preβ_1_-HDL that adsorbs free cholesterol efficiently from the cell membrane, is then transformed by the action of lecithin-cholesterol acyltransferase (LCAT), and is finally remodeled further by the activities of other plasma factors, such as hepatic lipase (HL), cholesterol ester transfer protein (CETP), and lipoprotein lipase (LPL), such that the nascent lipid-poor preβ_1_-HDL is converted into mature, cholesterol-rich, spherical HDL_2_, following the pathway of preβ_1_-HDL → preβ_2_-HDL → preβ_3_-HDL → HDL_3 _→ HDL_2 _[7-9]. The distribution of HDL subclasses can effectively reflect the efficiency of RCT and may directly impact the atherogenic progress.

Some investigators have reported the impaired conversion of preβ_1_-HDL to α-migrating HDL, thereby hampering RCT and likely contributing significantly to the high frequency of cardiovascular disease (CVD) occurrence in hemodialysis patients [10]; and other research has shown that concentrations of HDL_2 _are directly related to the efficiency in the clearance of triglyceride-rich fractions and exhibit the strongest negative correlation with both the advent and the extent of coronary atherosclerosis [11-13].

We have previously investigated the impact of plasma lipids together with their ratios on HDL subclass distribution and found that the particle size of HDL became smaller with the rise in plasma triglyceride (TG), TC, LDL-C levels, and TG/HDL-C along with total cholesterol (TC)/HDL-C ratios or with the fall of HDL-C levels [14-17]. Although the ratio of LDL-C/HDL-C shows promises as an attractive surrogate index of the atherogenicity of the plasma lipid profile, few data with regard to the association between the LDL-C/HDL-C ratio and the pattern of HDL subclass distribution are available. In this work, we evaluated the correlation between lipid variables, mainly the LDL-C/HDL-C ratio and the HDL subclass distribution profile in the Chinese population. HDL characterized by particle size could provide an important key to interpreting the effects of lipid parameters on HDL-related coronary heart disease (CHD) risk.

## Results

### Concentrations of plasma lipids, lipoproteins, apolipoproteins and the contents of HDL subclasses classified based on values of LDL-C to HDL-C

Characteristics of subgroups classified based on the LDL-C/HDL-C ratio were presented in Table 1. The concentrations of TG, TC, LDL-C and apoB-100 along with the ratios of TG/HDL-C, TC/HDL-C, and LDL-C/HDL-C increased successively; however, those of HDL-C and apoA-I decreased progressively with the elevation of the LDL-C/HDL-C ratio.

**Table 1:** Concentrations of plasma lipids, lipoproteins, and contents of HDL subclasses according to category of LDL-C/HDL-C ratio

	LDL-C/HDL-C≤2.3	2.3<LDL-C/HDL-C≤3.9	3.9<LDL-C/HDL-C≤4.6	LDL-C/HDL-C>4.6
**N**	166	261	48	35
**Age(y)**	56.0 ± 9.4	56.6 ± 9.6	57.4 ± 9.1	58.1 ± 8.6
**BMI(kg/m^2^)**	22.6 ± 2.8	23.9 ± 3.0	24.6 ± 2.9^a^*	25.0 ± 3.6^a^*
**TG(mmol/L)**	1.7 ± 0.2	2.1 ± 0.3^a†^	2.4 ± 0.5^a§b†^	2.8 ± 0.4^a§b§c†^
**TC(mmol/L)**	5.0 ± 0.3	5.6 ± 0.5^a†^	6.4 ± 0.8^a§b†^	6.9 ± 0.7^a§b§c†^
**LDL-C(mmol/L)**	2.5 ± 0.4	3.5 ± 0.5^a†^	4.3 ± 0.5^a§b†^	4.7 ± 0.7^a§b§c^*
**HDL-C(mmol/L)**	1.5 ± 0.2	1.2 ± 0.3^a§^	0.9 ± 0.2^a§b§^	0.7 ± 0.2^a§b§c†^
**TG/HDL-C**	1.6 ± 0.3	2.2 ± 0.4^a†^	2.8 ± 0.4^a§b†^	3.6 ± 0.5^a§b§c†^
**TC/HDL-C**	3.5 ± 0.7	5.0 ± 1.0^a§^	6.7 ± 0.8^a§b§^	8.2 ± 1.2^a§b§c§^
**LDL-C/HDL-C**	1.7 ± 0.5	3.0 ± 0.4^a§^	4.4 ± 0.4^a§b§^	5.6 ± 1.0^a§bc§§^
**ApoA-I (mg/L)**	1364.1 ± 114.2	1244.7 ± 109.2^a†^	1221.9 ± 105.8^a†^	1120.2 ± 116.9^a§b†c†^
**ApoB100(mg/L)**	803.6 ± 82.7	921.2 ± 89.0^a†^	1087.9 ± 90.3^a§b†^	1198.4 ± 93.2^a§b§c†^
**Pre_β1_-HDL(mg/L)**	81.2 ± 20.2	103.1 ± 25.7^a^*	121.9 ± 27.3^a†b^*	144.0 ± 35.9^a§b†c^*
**Pre_β2_-HDL(mg/L)**	59.6 ± 15.3	62.2 ± 19.3	67.1 ± 17.5	68.8 ± 22.0
**HDL_3c_(mg/L)**	72.2 ± 21.6	74.9 ± 25.1	78.9 ± 21.6	83.9 ± 20.1
**HDL_3b_(mg/L)**	140.8 ± 34.5	149.8 ± 37.8	173.4 ± 35.3^a†b^*	217.2 ± 33.1^a†b§c†^
**HDL_3a_(mg/L)**	274.9 ± 47.5	298.3 ± 58.3^a^*	314.7 ± 90.7^a†b^*	368.0 ± 92.1^a§b§c†^
**HDL_2a_(mg/L)**	314.3 ± 81.7	291.0 ± 50.6^a^*	250.8 ± 35.1^a§b†^	213.2 ± 24.8^a§b§c†^
**HDL_2b_(mg/L)**	387.3 ± 95.7	313.4 ± 79.7^a§^	261.7 ± 39.8^a§b§^	205.8 ± 29.2^a§b§c§^

Values are expressed as Mean ± S.D

*P < 0.05, ^†^*P *< 0.01, ^§^*P *< 0.001

^a^Significantly different from LDL-C/HDL-C≤2.3 group

^b^Significantly different from 2.3<LDL-C/HDL-C≤3.9 group

^c^Significantly different from 3.9<LDL-C/HDL-C≤4.6 group

The HDL subclass distribution profile was characterized by elevated contents of small-sized preβ_1_-HDL, HDL_3b _and HDL_3a_, whereas there was a reduction in the contents of large-sized HDL_2a _and HDL_2b _with the elevation of the LDL-C/HDL-C ratio.

### Characteristics of plasma apoA-I contents of HDL subclasses among subjects categorized by TG levels (2.26 mmol/L) and LDL-C/HDL-C ratio (3.5)

As Table 2 shows, the contents of preβ_1_-HDL and HDL_3a _were significantly lower, while the concentrations of HDL_2a _and HDL_2b _were significantly higher for subjects in the LDL-C/HDL-C < 3.5 group, compared with their counterparts in the LDL-C/HDL-C ≥3.5 group.

**Table 2:** The apoA-I contents of plasma HDL subclasses according to values of LDL-C/HDL-C along with TG levels

	LDL-C/HDL-C<3.5			LDL-C/HDL-C≥3.5		
	Total	TG<2.26mmol/L	TG≥2.26mmol/L	Total	TG<2.26mmol/L	TG≥2.26mmol/L
**N**	**386**	**249**	**137**	**124**	**50**	**74**
**Preβ_1_-HDL(mg/L)**	105.3 ± 20.2	80.4 ± 19.2	134.2 ± 25.5^b§^	120.8 ± 24.4^a^*	107.9 ± 21.9^c^*	127.8 ± 24.4^b^*
**Preβ_2_-HDL(mg/L)**	56.9 ± 10.8	56.6 ± 10.9	57.2 ± 13.4	59.9 ± 11.2	57.1 ± 11.4	465.0 ± 15.8
**HDL_3c_(mg/L)**	74.1 ± 19.7	73.4 ± 21.9	74.7 ± 21.0	75.0 ± 20.6	72.2 ± 19.8	76.9 ± 19.5
**HDL_3b_(mg/L)**	148.0 ± 25.9	145.2 ± 27.4	151.1 ± 28.6	150.0 ± 26.3	149.8 ± 24.4	163.8 ± 29.0
**HDL_3a_(mg/L)**	283.5 ± 34.6	241.5 ± 30.1	297.5 ± 70.8^b†^	309.2 ± 32.5^a^*	286.9 ± 66.9^c^*	336.3 ± 38.9^b§c†^
**HDL_2a_(mg/L)**	287.2 ± 29.0	320.8 ± 56.4	255.6 ± 31.0^b§^	243.7 ± 29.1^a†^	275.1 ± 29.1^c†^	210.8 ± 29.2^b*c†^
**HDL_2b_(mg/L)**	339.3 ± 41.7	374.7 ± 81.5	305.1 ± 35.1^b§^	260.2 ± 28.9^a§^	302.9 ± 78.9^c§^	221.1 ± 21.5^b†c^

Values are expressed as Mean ± S.D

*P < 0.05, ^†^*P *< 0.01, ^§^*P *< 0.001

^a^Compared with the Total subgroup within the LDL-C/HDL-C<3.5 group

^b^Compared with the TG<2.26mmol/L subgroup within the same LDL-C/HDL-C ratio group

^c^Compared with the LDL-C/HDL-C<3.5 subgroup within the same TG level group

Both in the LDL-C/HDL-C < 3.5 and LDL-C/HDL-C ≥3.5 groups, small-sized preβ_1_-HDL and HDL_3a _increased; however, large-sized HDL_2a _and HDL_2b _decreased more in the TG ≥2.26 mmol/L subgroup than in the corresponding TG < 2.26 mmol/L subgroup.

In addition, in the TG < 2.26 mmol/L and TG ≥ 2.26 mmol/L groups, the contents of preβ_1_-HDL (except TG ≥ 2.26 mmol/L group) and HDL_3a _increased significantly; in contrast, those of HDL_2a _and HDL_2b _decreased significantly in the LDL-C/HDL-C ≥ 3.5 group compared with in the LDL-C/HDL-C < 3.5 group.

### ApoA-I contents of plasma HDL subclasses according to plasma LDL-C and HDL-C concentrations among subjects

**Figure 1:**
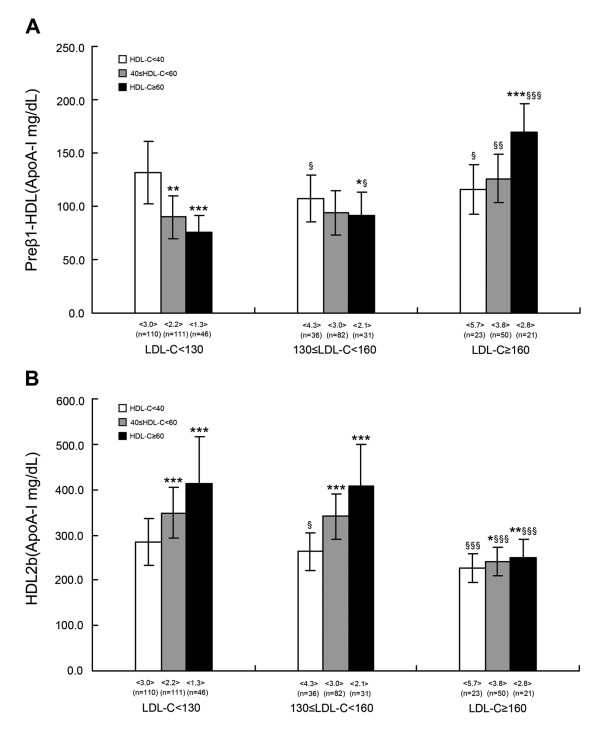
**The apoA-I contents of preβ_1_-HDL and HDL_2b _according to the concentrations of LDL-C and HDL-C**. Values are expressed as means. The values in the < > denote the LDL-C/HDL-C ratio; n: numbers; *P < 0.05, **P < 0.01, ***P < 0.001, compared with the HDL-C < 1.03 mmol/L subgroup within the same LDL-C group. ^§^*P *< 0.05, ^§§^*P *< 0.01, ^§§§^*P *< 0.001, compared with the same HDL-C subgroup within the LDL-C < 3.34 mmol/L group

As shown in Figure 1, compared to the low HDL-C (< 1.03 mmol/L) subgroup, preβ_1_-HDL decreased significantly but HDL_2b _increased significantly in the corresponding middle HDL-C (1.03-1.52 mmol/L) and high HDL-C (≥ 1.55 mmol/L) subgroups, both in desirable LDL-C (< 3.34 mmol/L) and borderline LDL-C (3.36-4.11 mmol/L) groups. On the contrary, in the high LDL-C (≥ 4.14 mmol/L) group, preβ_1_-HDL (high HDL-C subgroup) and HDL_2b _contents were markedly higher in the middle and high HDL-C subgroups than in the corresponding low HDL-C subgroup.

Moreover, in the borderline and high LDL-C groups, a reduction of preβ_1_-HDL and HDL_2b _contents was evident in the low HDL-C subgroup; in contrast, the contents of preβ_1_-HDL were obviously higher in the middle (high LDL-C group) and high HDL-C subgroups, while HDL_2b _was significantly lower for the middle and high HDL-C subgroups only in the high LDL-C group in comparison with the corresponding HDL-C subgroup in the desirable LDL-C group.

### Correlation coefficients between plasma lipids, ratios of lipids, and the apoA-I contents of HDL subclasses (controlling for TG)

After controlling for TG levels, correlation analysis results revealed that TC and LDL-C levels were positively correlated with preβ_1_-HDL and HDL_3 _(HDL_3c_, HDL_3b_, HDL_3a_) but negatively correlated with HDL_2a _(TC) and HDL_2b_. The ratios of TC/HDL-C and LDL-C/HDL-C were significantly positively correlated with preβ_1_-HDL but inversely correlated with HDL_2a _and HDL_2b_. Additionally, the HDL-C concentration was positively related to all HDL subclasses (Table 3).

**Table 3:** Correlation coefficients between plasma lipids along with lipids ratios and apoA-I contents of HDL subclasses

	Preβ_1_-HDL		Preβ_2_-HDL		HDL_3c_		HDL_3b_		HDL_3a_		HDL_2a_		HDL_2b_	
	Correlation Coefficient	*P*	Correlation Coefficient	*P*	Correlation Coefficient	*P*	Correlation Coefficient	*P*	Correlation Coefficient	*P*	Correlation Coefficient	*P*	Correlation Coefficient	*P*
**TC**	.395	.000	.110	.013	.217	.000	.289	.000	.110	.013	-0.117	.008	-.154	.001
**LDL-C**	.382	.000	.059	.184	.159	.000	.240	.000	.095	.032	.015	.743	-.228	.000
**HDL-C**	.150	.001	.131	.003	.164	.000	.141	.001	.099	.030	.187	.000	.270	.000
**TC/HDL-C**	.138	.002	-.036	.414	-.013	.768	.017	.699	.026	.555	-.174	.000	-.303	.000
**LDL-C/HDL-C**	.178	.000	-.034	.439	.008	.859	.043	.338	.034	.440	-.160	.000	-.301	.000

### Relationship of plasma apoA-I contents of HDL subclasses with levels of lipids, and ratios of lipids by multivariate stepwise regression analysis

To obtain a more complete understanding of the determinants of HDL subclass distribution, we performed stepwise multivariate regression analysis with the contents of all HDL subclass as dependent variables and the levels of TC, LDL-C, and HDL-C along with the ratios of TC/HDL-C and LDL-C/HDL-C as independent variables.

Table 4 shows that the contents of preβ_1_-HDL, HDL_3b_, HDL_3a _as well as HDL_2a _and HDL_2b _were significantly and independently predicted by the ratios of TC/HDL-C and LDL-C/HDL-C. Furthermore, TC was a significant independent negative predictor of the larger sizes of HDL_2a _and HDL_2b_; LDL-C was associated positively with the smaller sizes of HDL_3b _and HDL_3a_, in addition to the ratios of TC/HDL-C and LDL-C/HDL-C. The levels of TC, HDL-C, and LDL-C also made a significant contribution to preβ_1_-HDL, preβ_2_-HDL and HDL_3c_.

**Table 4:** Multiple stepwise regression correlation between contents of plasma HDL subclasses and concentrations of lipids along with ratios of lipids

		Unstandardized Coefficients		Standardized Coefficients	*t*	*p*
		B	Std. Error	Beta		
**Preβ_1_-HDL(mg/dL)**	**LDL-C/HDL-C**	29.39	2.97	.749	9.89	.000
	**TC**	29.32	2.83	.606	10.36	.000
	**TC/HDL-C**	15.75	1.79	.555	5.63	.000
	**HDL-C**	32.33	3.02	.292	3.58	.000
						
**Preβ_2_-HDL(mg/dL)**	**TC**	6.59	0.73	.282	3.67	.000
	**LDL-C**	4.49	0.51	.201	2.61	.009
						
**HDL_3c_(mg/dL)**	**TC**	6.15	0.37	.195	4.48	.000
	**HDL-C**	9.37	0.54	.130	2.98	.003
						
**HDL_3b_(mg/dL)**	**TC/HDL-C**	28.37	2.42	.831	2.72	.007
	**LDL-C/HDL-C**	46.86	4.14	.993	3.31	.001
	**LDL-C**	47.58	2.67	.854	3.24	.001
						
**HDL_3a_(mg/dL)**	**TC/HDL-C**	28.93	3.26	.535	4.62	.000
	**LDL-C/HDL-C**	42.56	4.01	.569	3.87	.000
	**LDL-C**	21.46	2.04	.243	3.05	.002
						
**HDL_2a_(mg/dL)**	**TC/HDL-C**	-26.60	3.77	-.566	-7.06	.000
	**LDL-C/HDL-C**	-14.36	1.56	-.221	-2.58	.000
	**TC**	-13.66	1.19	-.171	-3.60	.010
						
**HDL_2b_(mg/dL)**	**TC/HDL-C**	-55.52	5.16	-.801	-10.76	.000
	**LDL-C/HDL-C**	-41.08	3.61	-.429	-5.39	.000
	**TC**	-12.68	1.15	-.107	-2.44	.015

## Discussion

Increased levels of LDL-C and reduced levels of HDL-C are considered highly atherogenic. The LDL-C/HDL-C ratio possessed more prognostic value than LDL-C and HDL-C alone. In several studies, the LDL-C/HDL-C ratio has emerged as the best single lipid predictor of CHD risk [18]. Previous reports from the Monitored Atherosclerosis Regression Study (MARS) indicated that the LDL-C/HDL-C ratio was significantly associated with the progression of both low-grade and high-grade coronary artery lesions [19]. At the same time, the data showed that for every 1% reduction in the LDL-C/HDL-C ratio, there was a 31% reduction in the risk of a major cardiovascular event (MCVE) [20].

Total HDL in humans consists of several major and minor particle subclasses, where not all the HDL subclass particles have the same antiatherogenic potential. Minor variations in HDL subclass particle distribution might potentially cause larger variations in the coronary risk [21]. Some studies have demonstrated that both the severity and progression of CAD are related to the plasma concentration of individual HDL subclasses [22]. Thus, in this work, we mainly clarified the influence of the LDL-C/HDL-C ratio on HDL subclass distribution phenotype, which could have important implications.

According to the assessment from the Quebec Cardiovascular Study and calculated odds ratios for ischemic heart disease (IHD), increased IHD risk in the 3.9 to 4.6 and > 4.6 subgroups of the LDL-C/HDL-C ratio (odds ratio 3.4 and 3.7, respectively) compared with IHD odds ratio for the ≤ 2.3 along with the 2.3 to 3.9 LDL-C/HDL-C subgroups were 1.0 and 1.9, respectively [23]. Results from the current study revealed that, when accompanied by the elevation of the LDL-C/HDL-C ratio, the contents of small-sized preβ_1_-HDL, HDL_3a_, and HDL_3b _increased progressively but those of large-sized HDL_2a _and HDL_2b _decreased progressively. In the LDL-C/HDL-C ≤ 2.3 group, the contents of preβ_1_-HDL and HDL_2b _were 81.2 ± 20.2 and 387.3 ± 95.7 mg/dL, respectively. It is noteworthy that large-sized HDL_2a _and HDL_2b _occupied approximately 52.7% of the contents of HDL subclasses, the ratio of HDL_2b_/preβ_1_-HDL was approximately 4.8 in the LDL-C/HDL-C ≤ 2.3 group, and the distribution pattern of HDL subclass was consistent with the normolipidemic subjects in our previous report [15]. Compared with the LDL-C/HDL-C ≤ 2.3 group, an increase (27%, 50%, and 77%) in preβ_1_-HDL and a decrease (24%, 48%, and 88%) in HDL_2b _were found in the 2.3 < LDL-C/HDL-C ≤ 3.9, 3.9 < LDL-C/HDL-C ≤ 4.6 and LDL-C/HDL-C > 4.6 groups, respectively.

The description of HDL subclass distribution remodeling above might result from increased LDL-C and TG and decreased HDL-C levels. Many previous studies have indicated that enhanced CETP and HL activities are correlated with various levels of HDL-C[24,25]. CETP exchanges cholesteryl esters (CE) of HDL_2 _with TGs of very low density lipoprotein (VLDL) and LDL. The HDL-derived CE is removed from the circulation via the LDL receptor pathway. TGs in HDL are hydrolyzed by HL. The concerted action of CETP and HL converts larger HDL_2 _into smaller HDL_3 _and releases lipid-free apoA-I and/or preβ_1_-HDL [26-28]. On the other hand, a significant increase in TG levels with a rise in the LDL-C/HDL-C ratio was also observed in this study. It has been reported that high levels of TG are associated with impaired LPL and LCAT activities [29,30]. LPL is principally responsible for the hydrolysis of TGs in TG-rich lipoprotein (TRL) and the release of free fatty acids, transforming large TRL particles into smaller TG-depleted remnant lipoproteins. During this process, redundant surface lipid (FC and PL) and apo_s _are transferred from TRL to HDL_3_, resulting in the formation of HDL_2 _particles. LCAT plays an important role in the maturation of nascent to mature HDL catalyzed by the transfer of 2-acyl groups from lecithin to FC, generating CE and lysolecithin. Hydrophobic CE is retained in the HDL core, promoting the conversion of preβ_1_-HDL and HDL_3 _to HDL_2_. Thus, it has been suggested that a high LDL-C/HDL-C ratio is associated with low levels of large-sized HDL_2 _and generally with small-sized HDL particles.

Furthermore, several prospective studies [31,32] have revealed that a high LDL-C/HDL-C ratio combined with hypertriglyceridemia (HTG) is associated with the highest incidence of CHD. Castelli, *et al*. [33] observed that the average LDL-C/HDL-C ratio for people without CHD was below 3.4, while the ratio values for patients with excessive rates of CHD were at least 3.5. It is commonly thought that a desirable ratio of LDL-C/HDL-C is 3.5, which has been used as an indicator of coronary atherosclerosis [33]. Thus, in this context, we selected values of 3.5 for the LDL-C/HDL-C ratio and 2.26 mmol/L for TG, based on ATP-III guidelines, as critical cut-off points to examine the joint effect of the LDL-C/HDL-C ratio together with TG levels on the change in HDL subclass distribution.

The present results show increased contents of small-sized preβ_1_-HDL and HDL_3a _and decreased contents of large-sized HDL_2a_, and HDL_2b _in the LDL-C/HDL-C ≥ 3.5 group versus the LDL-C/HDL-C < 3.5 group, which indicated that the metabolism of HDL subclasses in the LDL-C/HDL-C ≥ 3.5 group was abnormal. In addition, there are 249 subjects, accounting for almost half of the total subjects in the TG < 2.26 mmol/L along with the LDL-C/HDL-C < 3.5 group; furthermore, the distribution of HDL subclass was also concordant with normolipidemic subjects from our previous study. On the contrary, the HDL subclass distribution in the TG ≥ 2.26 mmol/L together with LDL-C/HDL-C ≥ 3.5 group was characterized by elevated contents of small-sized preβ_1_-HDL, while large-sized HDL_2 _declined. That resulted in a drastic reduction of HDL_2b_/preβ_1_-HDL (4.7 down to 1.7) and the percentage of the mature HDL_2a _and HDL_2b _(53.8% down to 35.7%), compared to the TG < 2.26 mmol/L group along with the LDL-C/HDL-C < 3.5 group. It indicated that HDL metabolic disorders and HDL maturation are blocked in the TG ≥ 2.26 mmol/L along with the LDL-C/HDL-C ≥ 3.5 group. At the same time, it is reported that the most common hyperlipidemia in the Chinese population is characterized by elevated TG levels (e.g., HTG), which accounts for about 61% of total hyperlipidemia [8]. In this regard, it is important to point out that applying the LDL-C/HDL-C ratio to estimate the risk of CHD should be combined with the effect of individual TG levels, particularly in the Chinese population.

The current NCEP (ATP-III) guidelines recommend specific target levels of LDL-C and HDL-C for determining CVD risk and assessing the effectiveness of lipid-lowering therapies. The interaction of concentrations of LDL-C and HDL-C and values of the LDL-C/HDL-C ratio with HDL subclass distribution was also investigated in our study. We observed in subjects with low HDL-C levels a marked increased in the LDL-C/HDL-C ratio (3.0, 4.3, and 5.7), and the contents of HDL_2b _decreased progressively and significantly, and all three remained at lower concentrations (284.6, 263.4 and 227.4 mg/L); however, contents of preβ_1_-HDL stayed at higher concentrations (131.7, 107.6, and 115.3 mg/L) in the desirable LDL-C (< 3.34 mmol/L), borderline LDL-C (3.36-4.11 mmol/L) and high LDL-C (≥ 4.14 mmol/L) groups, respectively, with elevation of LDL-C levels. These findings suggest that in low concentrations of HDL-C, abnormalities in the HDL particle profile occur regardless of whether the LDL-C levels increased or not. Moreover, other studies have demonstrated that low HDL-C increased the risk of CVD, even when LDL-C was normal or without marked elevation [2,34].

On the other hand, we also noted that in high LDL-C level (≥ 4.14 mmol/L) subjects who had a relatively low LDL-C/HDL-C ratio (2.8), the distribution in the HDL subclass was substantially altered and characterized by increased small-sized preβ_1_-HDL (169.5 mg/L) and drastically falling large-sized HDL_2b _(251.1 mg/L), which resulted in an extreme low value of HDL_2b_/preβ_1_-HDL (1.4) in spite of the higher concentrations of HDL-C. The opposite of this pattern was observed in the borderline LDL-C (3.36-4.11 mmol/L) and desirable LDL-C (< 3.34 mmol/L) groups, and the subjects in these two groups had significantly higher HDL_2b_/preβ_1_-HDL values (4.4 and 5.4, respectively) and maintained the distribution of the HDL subclass in a normal or near-normal profile. Specific HDL subclasses are involved in certain steps of RCT, and a low HDL_2b_/preβ_1_-HDL ratio is a signature pattern of disturbed HDL metabolism and RCT, implying that higher concentrations of HDL-C do not correct the abnormal HDL subclass distribution for subjects with high levels of LDL-C (≥ 4.14 mmol/L). Furthermore, it is interesting to note that there was a significant reduction of the HDL_2b_/preβ_1_-HDL ratio (2.1 and 1.4), although there was a lower ratio of LDL-C/HDL-C (3.0 and 2.8) according to the Castelli risk indices (< 3.5) in both the desirable LDL-C-low HDL-C and high LDL-C-high HDL-C groups. The correlation analysis results also support the above observations. The levels of TC and LDL-C together with the ratios of TC/HDL-C and LDL-C/HDL-C were positively correlated with preβ_1_-HDL, while there was the inverse relationship with HDL_2b_; the plasma HDL-C level was positively related to preβ_1_-HDL and HDL_2b _in both univariate and stepwise regression analyses (Tables 3 and 4). These findings together suggest that the levels of LDL-C and HDL-C along with the values of LDL-C/HDL-C should be considered simultaneously while assessing the HDL subclass metabolism and determining CVD risk.

It is worthwhile to point out that diets high in carbohydrates are prevalent in China, resulting in increased concentrations of plasma glucose and, thus, high insulin levels. Hyperinsulinemia stimulates the production and secretion of TG and VLDL, which lead to relatively higher TG and lower HDL-C levels. The typical high TG and low HDL-C in the Chinese population are accompanied by the abnormal distribution of the HDL subclass. Based on the abovementioned, the elevated TG along with reduced HDL-C levels might be the cause of the alteration of the HDL subclass distribution in the Chinese population. Unlikeness, Western populations usually have a high saturated fat diet (Western type of diet), and an increase in dietary fat will elevate plasma TC and LDL-C concentrations [35-37]. Also, levels of TC and LDL-C [24,38] showed a positive correlation with CETP and HL activities, which favor the formation of small-sized HDL particles. The concentration of plasma lipids could be an important factor impacting the HDL subclass distribution in the Western population.

## Conclusions

Overall, the results of this study suggest that a general shift toward smaller-sized HDL was accompanied by an increase of the LDL-C/HDL-C ratio, suggesting that the maturation process of HDL was blocked. The shift mentioned above would be more obvious with a high LDL-C/HDL-C ratio and elevated TG levels. The HDL subclass distribution can be effectively normalized by a high HDL-C concentration at desirable borderline LDL-C levels (< 4.11 mmol/L). The influence of higher LDL-C levels on the HDL subclass, however, cannot be counteracted by higher HDL-C levels.

## Methods

### Subjects

This protocol was approved by West China Hospital, Sichuan University (Chengdu 610041, China). Five hundred ten subjects (323 men; mean age, 56.2 ± 9.0 years; 187 women; mean age, 56.5 ± 9.2 years) were recruited to participate in a study examining plasma lipid and apo concentrations at West China Medical Center, Sichuan University. Exclusion criteria were the following: (1) the presence of nephrosis, diabetes mellitus, hypothyroidism, or hepatic impairment; (2) the presence of a major cardiovascular event (myocardial infarction, severe or unstable angina pectoris, and surgery) or stroke; (3) taking lipid-altering medications; or (4) a history of alcohol abuse and smoking cigarettes. Informed consent was obtained from each subject upon entry into the study population. This study protocol was approved by the ethics committee.

To study the relationship between the LDL-C/HDL-C ratio and HDL subclass distribution, we divided these subjects into four subgroups using the cut-off points of less than 2.3, 2.3 to 3.9, 3.9 to 4.6, and higher than 4.6, as suggested by the Quebec Cardiovascular Study [23]. The average LDL-C/HDL-C ratio was 3.5 or higher [33] and has been suggested as a value indicating increased CHD risk. We also assessed the joint effect of the LDL-C/HDL-C and TG levels on the HDL subclass distribution based on 3.5 for LDL-C/HDL-C and 2.26 mmol/L for TG as the cut-off points. The alteration of the LDL-C and HDL-C levels associated with the phenotype of HDL subclass distribution was also investigated in the current work. The reference levels of the plasma lipids were defined by following guidelines from the Adult Treatment Panel III (ATP-III) of the National Cholesterol Education Program (NCEP) [39], that is: desirable LDL-C (< 130 mg/dL; 3.34 mmol/L), borderline LDL-C (130-160 mg/dL; 3.36-4.11 mmol/L), high LDL-C (≥ 160 mg/dL; 4.14 mmol/L), low HDL-C (< 40 mg/dL; 1.03 mmol/L), middle HDL-C (40-60 mg/dL; 1.03-1.55 mmol/L), and high HDL-C (≥ 60 mg/dL; 1.55 mmol/L).

### Specimens

Whole blood specimens were drawn after a 12 h overnight fast into EDTA-containing tubes. Plasma was separated within 1-2 h. Plasma was stored at 4°C and used for lipid and apo analyses within 24 hours. An aliquot of plasma was stored at -70°C for the determination of HDL subclasses.

### Plasma lipid and apolipoprotein analyses

Plasma TG, TC and HDL-C concentrations were measured by the standard technique. TC and TG were determined with enzymatic kits (Beijing Zhongsheng Biotechnological Corporation, Beijing, and People's Republic of China). The HDL-C was determined after precipitation of the apoB-containing lipoproteins by phosphotungstate/magnesium chloride [40]. LDL-C was calculated using the Friedwald formula (TG < 4.52 mmol/L) [41]. When plasma TG was at least 4.52 mmol/L, LDL-C was determined following the precipitation method with polyvinylsulfate (enzymatic kits). Plasma apoA-I, B-100, C-II, and C-III were determined by radial immunodiffusion methods [42] using kits developed at the Apolipoprotein Research Laboratory, West China Medical Center, Sichuan University. The intraassay coefficient of variation for apo concentrations was between 2.1% and 4.8%; the interassay coefficient of variation was 3.5% to 7.9% [43].

### High density lipoprotein cholesterol subclass analysis

ApoA-I-containing HDL subclasses were measured by nondenaturing two-dimensional gel electrophoresis associated with the immunodetection method, as described previously [7]. Briefly, 10 μl of plasma was first separated by charge on 0.7% agarose gel into preβ and α mobility particles. In the second dimension, the two fractions of HDL were further separated according to size by 2-30% nondenaturing polyacrylamide gradient gel electrophoresis. Western blotting was conducted to determine HDL subclasses after electrophoresis using horseradish peroxidase (HRP)-labeled goat anti-human apoA-I immunoglobulin G (IgG). The HDL particle sizes were calibrated using a standard curve that included bovine serum albumin, ferritin and thyroglobulin (Pharmacia, Uppsala, Sweden). The calculation of the relative percentage of each HDL subclass was based on the density of the electrophoresis spots. Then, the apoA-I contents (in milligrams per liter) of the HDL subclasses were calculated by multiplying the percentage of each subclass by the plasma total apoA-I concentrations. The interassay coefficients of variation of the relative concentration of preβ_1_-HDL, preβ_2_-HDL, HDL_3c_, HDL_3b_, HDL_3a_, HDL_2a_, and HDL_2b _in the plasma sample were 9.4%, 9.8%, 4.9%, 6.2%, 7.3%, 11.1% and 7.9%, respectively (n = 5).

### Statistical analysis

All statistical analyses were performed using the statistical package SPSS Version 13.0 (SPSS, Chicago, IL). Data in the tables are expressed as mean ± S.D. The Duncan post-hoc test was used in situations in which a significant group (one-way analysis of variance) effect was observed. Pearson correlation coefficients were calculated to quantitatively analyze the associations among variables adjusted for TG concentration. Multivariate stepwise regression analyses were computed to estimate the independent contribution of metabolic variables to the distribution of HDL subclasses. In all comparisons, *P *less than 0.05 (2-sided) represented a statistically significant difference.

## Conflict of interest statement

The authors declare that they have no competing interests.

## Authors' contributions

LT participated in the design of study and manuscript preparation along with editing. YHL performed data and statistics analysis. YQ conceived of the study, and helped to review the manuscript. SYL participated in drafted the manuscript. YHX performed the data acquisition and analysis. MDF participated in study concepts and critical review of study proposal. All authors read and approved the final manuscript.
